# Classification of Nitrogen-Efficient Wheat Varieties Based on UAV Hyperspectral Remote Sensing

**DOI:** 10.3390/plants14131908

**Published:** 2025-06-20

**Authors:** Yumeng Li, Chunying Wang, Junke Zhu, Qinglong Wang, Ping Liu

**Affiliations:** 1 Shandong Engineering Research Center of Agricultural Equipment Intelligentization, Shandong Key Laboratory of Intelligent Production Technology and Equipment for Facility Horticulture, College of Mechanical and Electronic Engineering, Shandong Agricultural University, Tai’an 271018, China; 2022010106@sdau.edu.cn (Y.L.); chunyingwang@sdau.edu.cn (C.W.); 2023010106@sdau.edu.cn (Q.W.); 2 State Key Laboratory of Wheat Improvement, Shandong Agricultural University, Tai’an 271018, China; 3 School of Agricultural and Food Engineering, Shandong University of Technology, Zibo 255100, China; zhunjunke@sdut.edu.cn

**Keywords:** UVA, hyperspectral remote sensing, variety classification, machine learning, ensemble learning

## Abstract

Aiming at tackling the challenges of traditional classification methods, which are labor-intensive, time-consuming, and inefficient, a nitrogen-efficient wheat variety classification method using support vector machine-extreme gradient boosting (SVM-XGBoost) based on unmanned aerial vehicle (UAV) hyperspectral remote sensing was proposed in this study. First, eight agronomic indicators closely related to wheat nitrogen efficiency were analyzed using t-SNE dimensionality reduction and hierarchical clustering, enabling the classification of 12 wheat varieties into nitrogen-efficient and nitrogen-inefficient varieties under different nitrogen stress conditions. Second, a hyperspectral feature band selection method based on least absolute shrinkage and selection operator-competitive adaptive reweighted sampling (Lasso-CARS) was employed using hyperspectral canopy data collected during the wheat heading stage with an UAV to extract feature bands relevant to nitrogen-efficient wheat classification. This approach aimed to mitigate the impact of high collinearity and noise in high-dimensional hyperspectral data on model construction. Furthermore, the SVM-XGBoost method integrated the extracted feature bands with the support vectors and decision function outputs from the preliminary SVM classification. It then leveraged XGBoost to capture nonlinear relationships and construct the final classification model using gradient-boosted trees, achieving intelligent classification of nitrogen-efficient wheat varieties. The model also selected nitrogen fertilization strategies based on the characteristics of different wheat varieties. The results demonstrated robust performance under low, high, and no nitrogen stress, with average overall accuracies of 74%, 83%, and 70% (Kappa coefficients: 0.67, 0.80, and 0.48), respectively. This study provided an efficient and accurate UAV hyperspectral remote sensing-based method for nitrogen-efficient wheat variety classification, offering a technological foundation to accelerate precision breeding.

## 1. Introduction

Wheat, as one of the world’s three major food crops, plays a critical role in global food production, providing a solid foundation for human survival and development [1,2]. With its wide planting range, high yield potential, and rich nutritional value, wheat continues to supply essential food resources for humanity [3,4]. Nitrogen is a key nutrient in wheat growth and yield formation. Proper nitrogen fertilizer application can promote healthy wheat development, ultimately increasing yield and optimizing quality [5]. However, with the development of the seed industry, the number of wheat varieties with different characteristics has increased. Failure to adjust the nitrogen fertilizer application rate after changing the variety of wheat being cultivated may result in nitrogen deficiency or excess in the crop, subsequently affecting yield and contributing to environmental pollution [6,7]. Therefore, breeding nitrogen-efficient varieties of wheat capable of nitrogen efficiency and adjusting nitrogen fertilizer application strategies not only improves wheat yield and quality, but also increases nitrogen use efficiency and optimizes nitrogen fertilizer application [8,9]. This method can solve the problem of mismatch between wheat varieties and nitrogen fertilizer application, reduce environmental pollution, and promote the sustainable and healthy development of agricultural production. It is of great significance to the sustainable development of the wheat industry.

Research has shown that wheat varieties exhibit differences in nitrogen absorption and utilization under varying growing conditions [10,11,12]. Wheat varieties that maintain normal growth and yield in low-nitrogen environments as well as those that grow and yield more in high nitrogen environments can be categorized as having high-nitrogen efficiency [13]. Nitrogen efficiency in wheat is a complex trait, often evaluated through multiple phenotypic indicators [14,15]. Zhang et al. identified two high-yield and nitrogen-efficient varieties from major wheat cultivars in the Huanghuaihai region, considering both yield and nitrogen efficiency [16]. Guo et al. demonstrated a highly significant positive correlation between wheat yield at maturity and nitrogen accumulation, nitrogen use efficiency, and nitrogen responsiveness, proposing these as key evaluation metrics for wheat nitrogen efficiency [17]. However, evaluating whether a wheat variety is nitrogen efficient is a complex process that involves multiple aspects, such as wheat yield and growth performance. Despite the differences in classification criteria for nitrogen efficiency across various studies at present, clustering methods are ultimately employed for the final classification. Therefore, it is an effective and feasible approach to utilize phenotypic changes, such as wheat yield and nitrogen accumulation, as well as agronomic indices like nitrogen use efficiency under different nitrogen stress conditions, as indicators for evaluating nitrogen efficiency in wheat. Furthermore, classifying wheat varieties into nitrogen-efficient and nitrogen-inefficient varieties under different nitrogen stress conditions using clustering is a practical method. These traditional methods for classifying nitrogen-efficient wheat varieties rely on metrics such as yield and nitrogen accumulation, which are typically obtained through destructive sampling or manual testing after harvest. Although accurate, these methods are inefficient, labor-intensive, and time-consuming, particularly in large-scale field breeding environments with numerous varieties [18]. Moreover, they can only be applied after wheat harvest, limiting their timeliness. Therefore, there is an urgent need to develop a large-scale, high-throughput method to achieve accurate classification of nitrogen-efficient wheat varieties. Machine learning, with its ability to automatically extract key features from vast amounts of data and identify complex patterns and relationships, is the best choice to achieve this goal. Additionally, machine learning possesses powerful computational capabilities and efficient parallel processing abilities, enabling rapid processing of large-scale datasets and significantly improving classification efficiency. Moreover, the excellent adaptability and generalization capabilities of machine learning allow it to maintain stable performance across different environments and conditions, thereby enhancing the accuracy of classification.

UAV remote sensing technology, as a recently developed and highly integrated interdisciplinary innovation, has demonstrated immense application potential in modern agriculture [19,20,21]. This technology significantly enhances the speed of collecting crop canopy spectral data. Additionally, it enables non-destructive and accurate identification of key information such as crop growth status [22], physiological and biochemical traits [23], and comprehensive phenotypes [24]. Jiang et al. utilized UAV-based multispectral imagery to predict plant dry matter, nitrogen accumulation, and nitrogen nutrition index values, enabling the detection of wheat nitrogen status [25]. In contrast, research focusing on accurately evaluating crop nitrogen efficiency is relatively limited. Dong et al. constructed estimation models for nitrogen-related traits, such as nitrogen use efficiency, based on UAV multispectral imagery. They performed clustering analysis on the predicted values to identify nitrogen-efficient wheat varieties, demonstrating the strong potential of UAV remote sensing for wheat variety classification [26].

However, despite demonstrating certain potential in agricultural applications, existing multispectral remote sensing technology is constrained by its ability to capture only specific broad-band information. This technical characteristic makes it difficult to comprehensively and precisely detect spectral differences across all wavelengths of the wheat canopy, thereby limiting its effectiveness in precision agriculture, particularly within the niche area of classifying nitrogen-efficient wheat varieties [27]. Specifically, multispectral remote sensing typically provides information from only a few discrete bands. Although these bands can reflect certain specific physiological or biochemical characteristics, they fail to adequately capture the subtle variations in the spectral features of the wheat canopy, especially those closely related to nitrogen absorption and utilization efficiency. Existing research on the classification of nitrogen-efficient wheat varieties primarily relies on traditional field experiments and laboratory analysis methods, such as assessing nitrogen efficiency by measuring indicators like the leaf area index, nitrogen accumulation, and yield of wheat. Although these methods can yield relatively accurate results under specific conditions, they have significant limitations. First, these approaches often require extensive destructive field sampling and laboratory analysis, which are not only time-consuming and labor-intensive but also costly, making them difficult to apply on a large scale in breeding and production. In contrast, hyperspectral remote sensing technology offers a new perspective for classifying nitrogen-efficient wheat varieties due to its capability to acquire hyperspectral data of the wheat canopy. Hyperspectral remote sensing can continuously record reflectance spectral information from the wheat canopy across hundreds of consecutive bands. This spectral information not only encompasses physical structural details of the wheat canopy but also contains a wealth of biochemical composition information, particularly that related to nitrogen absorption and utilization efficiency [28,29]. Therefore, through hyperspectral remote sensing technology, we can gain a more comprehensive understanding of the spectral characteristics of the wheat canopy and subsequently assess its nitrogen efficiency more accurately. However, despite the notable advantages of hyperspectral remote sensing technology in classifying nitrogen-efficient wheat varieties, relevant research remains limited.

To address the issues of high labor intensity, time-consuming nature, and low efficiency associated with traditional classification methods for nitrogen-efficient wheat varieties, this study proposes a nitrogen-efficient wheat variety classification method utilizing UAV hyperspectral remote sensing technology. Based on the clustering results of agronomic indicators and canopy hyperspectral data, the method employs the Lasso-CARS algorithm to effectively extract a combination of characteristic bands under both low- and high-nitrogen stress conditions. This approach mitigates the impact of noise and redundant variables, thereby enhancing classification accuracy. Furthermore, the proposed classification method integrates SVM with XGBoost, fully leveraging the advantages of both algorithms to construct a robust and highly accurate classification model. The model effectively circumvents potential issues such as overfitting that may be inherent in single-model architectures, achieving efficient and accurate classification of nitrogen-efficient wheat varieties. This research provides technical support for the application of UAV hyperspectral remote sensing technology in wheat breeding and production.

## 2. Results

### 2.1. Subsection

The differences in eight agronomic traits, including WY, ANA, WDMM, TGW, UNE, AENF, NFUE, and NT, were analyzed for twelve different wheat varieties. As shown in Table 1 and Table 2, under low-nitrogen stress, the coefficient of variation for the eight indicators ranged from 0.06% to 13.37%, with the largest variation coefficient for WY (13.37%) and the smallest for NT (0.06%). Under high-nitrogen stress, the coefficient of variation for the eight indicators ranged from 0.06% to 20.00%, with the largest variation coefficient for AENF (20.00%) and the smallest for NT (0.06%). The eight indicators exhibited varying degrees of difference among the twelve wheat varieties, which can serve as a basis for evaluating the nitrogen efficiency of wheat varieties.

### 2.2. Determination and Analysis of Nitrogen Stress-Tolerant Wheat

**Figure 1:**
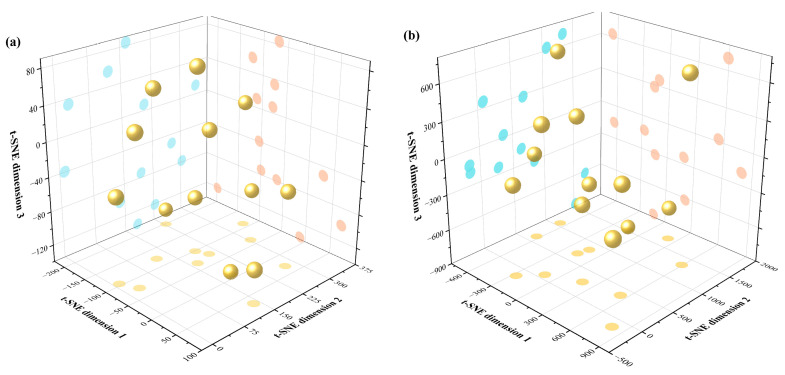
Three-dimensional t-SNE plots of different wheat varieties. (**a**) Dimensionality reduction results under low-nitrogen stress; (**b**) Dimensionality reduction results under high-nitrogen stress. Note: The blue, yellow, and red circles on the planes respectively represent the projections of the dimensionality reduction results onto the planes of dimension 2 & 3, dimension 1 & 2, and dimension 1 & 3.

t-SNE is an effective nonlinear dimensionality reduction technique. It calculates the conditional probabilities between all data points in the high-dimensional space as a representation of similarity and measures the degree of similarity between data points using a Gaussian distribution to create corresponding probability distributions for the data points based on the results. Using this as a reference, a similar probability distribution was created in the low-dimensional space, and the gradient descent method was used to iteratively adjust the positions of the data points in the low-dimensional space until the optimal low-dimensional representation of each data point was obtained. Outliers maintained their uniqueness after t-SNE dimensionality reduction. This aided in the exploration and visualization of the structure of high-dimensional data and the identification of clusters that may not be apparent in the original data. Dimensionality reduction was performed separately on the wheat agronomic indicators after standardization under high-nitrogen stress and low-nitrogen stress environments. The results of the dimensionality reduction are shown in Figure 1.

Due to the different definitions of nitrogen efficiency in various nitrogen stress environments, wheat varieties from different nitrogen stress trials were classified separately. Hierarchical clustering was performed based on the spatial relationships obtained after t-SNE dimensionality reduction, and the varieties were grouped into two clusters. Initially, each sample point in the dataset was treated as a separate cluster, and the similarity between each pair of clusters was calculated. The two clusters with the shortest distance were then merged into one, and the similarity between the new cluster and the existing clusters was recalculated. This process was repeated until all data points were clustered into two groups. To retain the local similarity of the comprehensive phenotypes of wheat varieties after dimensionality reduction, the complete linkage method was used to calculate the similarity between the clusters.

**Figure 2:**
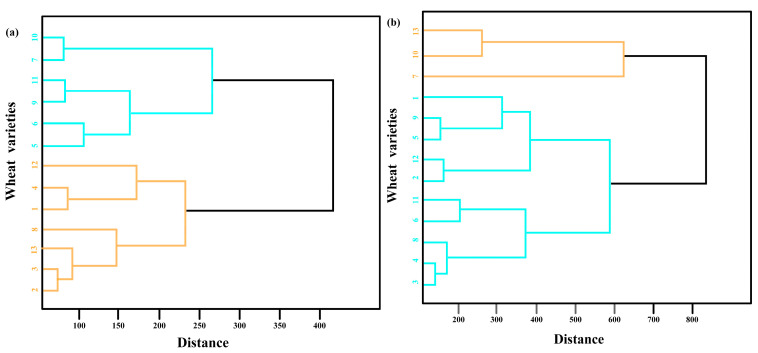
Schematic diagram of hierarchical clustering. (**a**) Clustering results under low-nitrogen stress; (**b**) Clustering results under high-nitrogen stress. Note: The orange and blue colors represent nitrogen-efficient and nitrogen-inefficient wheat varieties respectively.

The hierarchical clustering results are shown in Figure 2. For the wheat planted in low-nitrogen stress environments, Jimai 22 (labeled 13) was used as the standard. The varieties were classified into six nitrogen-efficient varieties (labeled 1, 2, 3, 4, 8, and 12), and six nitrogen-inefficient wheat varieties. For the wheat planted in high-nitrogen stress environments, Shannong 28 (labeled 13), a nitrogen-efficient variety, was used as the standard. The varieties were classified into two nitrogen-efficient varieties (labeled 7 and 10) and ten nitrogen-inefficient wheat varieties.

**Figure 3:**
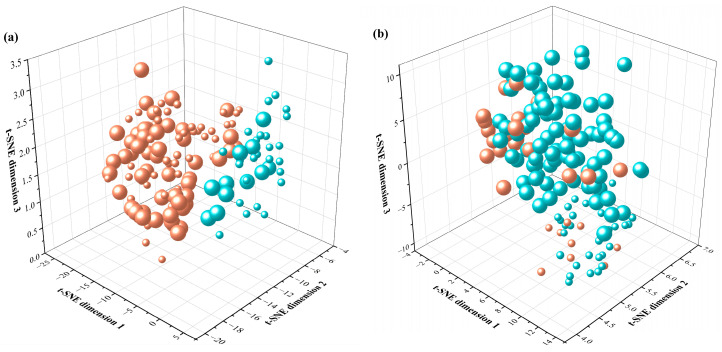
Schematic diagram of classification results after using t-SNE dimensionality reduction and hierarchical clustering based on hyperspectral reflectance. (**a**) Classification results under low-nitrogen stress; (**b**) Classification results under high-nitrogen stress. Note: The orange and blue colors of the spheres respectively represent nitrogen-efficient and nitrogen-inefficient wheat varieties obtained through hierarchical clustering. The large and small sizes of the spheres represent nitrogen-inefficient and nitrogen-efficient wheat varieties in the actual classification, respectively.

Hyperspectral data of wheat canopy from various plots were obtained using a hyperspectral UAV. The reflectivity of five locations within each plot was extracted. The CV for reflectivity across various spectral bands was calculated, with 88% of the spectral bands having a CV greater than 10%, indicating varying degrees of difference that could be utilized for classifying nitrogen-efficient wheat varieties. These data were used as input for hierarchical clustering. The results are shown in Figure 3.

Orange and blue represent nitrogen-efficient and nitrogen-inefficient wheat varieties, respectively, as determined by clustering. The size of the markers indicated actual classifications, with larger markers representing nitrogen-inefficient and smaller markers representing nitrogen-efficient varieties. For wheat grown under high-nitrogen stress environments, the overall classification accuracy for nitrogen-efficient varieties was 69%. Similarly, for wheat grown under low-nitrogen stress environments, the overall classification accuracy was 56%. Although there were noticeable differences in canopy hyperspectral reflectance between nitrogen-efficient and nitrogen-inefficient wheat under both high- and low-nitrogen stress conditions, the high dimensionality of hyperspectral data introduced issues such as redundancy and noise, which adversely affected classification accuracy. Additionally, hierarchical clustering had significant limitations when handling large datasets, including increased computational costs and reduced time efficiency. A critical drawback of hierarchical clustering was the irreversibility of merging or splitting steps during the clustering process. Once a step was completed, it could not be undone, potentially leading to incorrect decisions during clustering. The presence of outliers and irregular cluster boundaries in canopy reflectance data further reduced the accuracy of clustering in this context.

### 2.3. Classification of Nitrogen-Efficient Wheat Varieties Based on Hyperspectral Feature Bands

**Figure 4:**
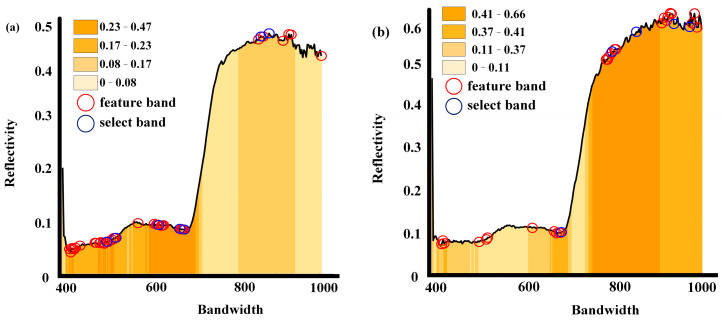
Feature band selection results. (**a**) Feature bands selected under low-nitrogen stress; (**b**) Feature bands selected under high-nitrogen stress.

To eliminate the effects of redundant bands and noise, improve model accuracy, and reduce runtime, this study proposed a hyperspectral feature band extraction method based on the Lasso-CARS algorithm. First, the dataset, which included 224 bands of canopy regions from 240 plots of 12 wheat varieties grown in both low- and high-nitrogen stress environments, along with their corresponding wheat variety classifications, was used as input. After initializing the Lasso model, training was conducted to learn the relationships between each feature band and the classification targets. Cross-validation was then performed to evaluate the performance of the Lasso model with different regularization parameters (alpha) to obtain the optimal alpha value. The coefficients of the Lasso model at the optimal alpha value were then determined. As the Lasso model tended to shrink the coefficients of unimportant features to zero, the subset of non-zero coefficients corresponded to the feature bands that were most strongly related to the classification results after removing high collinearity and redundant bands. As shown in Figure 4, these feature bands were represented by red circles on the reflectance curve.

Additionally, to demonstrate the accuracy of Lasso feature selection, the correlations between each band and the different wheat varieties were computed. The results were labeled from light to dark according to the quartiles from high to low and displayed in the lower region of the hyperspectral curve. Figure 4a showed the 42 feature bands selected under low-nitrogen stress conditions, covering most of the high-correlation band intervals. Three concentrated feature band intervals (400 nm–540 nm, 600 nm–660 nm, 850 nm–900 nm) overlapped with the high-correlation regions. Figure 4b shows the feature band subset selected under high-nitrogen stress conditions, including 35 feature bands. Compared to Figure 4a, the distribution was more sparse, but it still covered most of the high-correlation band intervals. The feature bands were more concentrated in the reflectance peak intervals and the 700 nm–900 nm range.

After the preliminary feature selection using Lasso, the feature band subset was further optimized using the CARS method. CARS simulated the variable importance analysis in the PLS model and combined Monte Carlo sampling with an adaptive weight adjustment mechanism to gradually eliminate redundant feature bands and retain the key bands with higher informational value. Specifically, CARS generated subsets using random sampling and built regression models to calculate the importance values of variables. It dynamically adjusted the feature weights using a weighted mechanism, gradually converging to the optimal feature band set after multiple iterations. This process effectively removed noise and redundant bands while avoiding the interference of highly correlated variables. As a result, seven feature bands were extracted under low-nitrogen stress conditions (497.71 nm, 513.64 nm, 601.78 nm, 636.78 nm, 652.99 nm, 836.22 nm, 841.75 nm), and five feature bands were extracted under high-nitrogen stress conditions (682.79 nm, 794.84 nm, 838.98 nm, 936.38 nm, 975.65 nm). The finally selected feature bands were indicated by the blue circles on the reflectance curve in Figure 4.

In this study, 120 canopy regions from different small plots under low- and high-nitrogen stress environments were used as objects for classification. The feature subsets and corresponding classification categories obtained were used as inputs. The SVM-XGBoost method was employed to construct the classification model for nitrogen-efficient wheat varieties. Additionally, comparative experiments were conducted with the RF, SVM, XGBoost, and Adaboost algorithms using the same dataset and evaluation metrics. The experimental results are shown in Table 3.

Under low-nitrogen stress conditions, significant differences in the classification performance of various algorithms were observed. The SVM-XGBoost algorithm performed the best, with an OA of 74% and a Kappa coefficient of 0.67, significantly outperforming other algorithms. This indicated that it had the strongest adaptability to low-nitrogen stress spectral features. In contrast, the SVM algorithm showed the poorest performance with an OA of 55% and a Kappa coefficient of 0.55, demonstrating limited ability to handle complex spectral data under low-nitrogen stress. The other algorithms had moderate performance. Overall, the SVM-XGBoost algorithm showed clear superiority under low-nitrogen stress conditions.

Under high-nitrogen stress conditions, the classification performance of all algorithms generally improved. The SVM-XGBoost algorithm remained the best performer, with an OA of 83% and a Kappa coefficient of 0.8, further demonstrating its high adaptability to high-nitrogen stress spectral features. It was clear that under high-nitrogen stress, all algorithms benefited from the increased spectral data discriminability, but the SVM-XGBoost performance remained the leading one.

In terms of average performance, the SVM-XGBoost algorithm consistently outperformed the others, with an OA of 76% and a Kappa coefficient of 0.57, making it the best performing method. In particular, compared to the original Adaboost algorithm, the average OA and Kappa coefficient showed significant improvements. Additionally, the classification performance under high-nitrogen stress was better than that under low-nitrogen stress. This improvement could be due to the wheat varieties used in this experiment being preliminarily selected as potential nitrogen-efficient varieties. Under low-nitrogen stress, the overall nitrogen absorption and utilization efficiency increased for all varieties, resulting in smaller differences between them. However, under high-nitrogen stress, higher nitrogen fertilizer application fully revealed the potential of these varieties. Nitrogen-efficient varieties under high-nitrogen stress exhibited significantly higher nitrogen absorption and utilization efficiency than other varieties, leading to more pronounced differences in canopy nitrogen content and other phenotypic traits, which improved the classification results.

Considering that different feature subset extraction methods yield different results, comparisons were made using the full-band subset, CARS- and Lasso-selected feature subsets, and the final feature subset obtained using the Lasso-CARS algorithm as inputs. These subsets were tested under both low- and high-nitrogen stress conditions using the constructed classification model. The model evaluation metrics are shown in Table 4.

There were significant differences in the classification performance of the methods under low-nitrogen stress conditions. The performance of the full-waveband method was the weakest. This indicated that the full-band method did not fully utilize the spectral information under low-nitrogen stress. The CARS algorithm improved the classification performance under low-nitrogen stress through feature selection. The OA of the Lasso algorithm was the same as that of the full-band method, but its Kappa coefficient increased, which indicated that consistency had been improved. Notably, the Lasso-CARS algorithm performed the best with a 74% OA and a 0.67 Kappa coefficient, which was significantly better than the other methods. This trend suggested that the Lasso-CARS algorithm based on spectral feature selection was more effective in extracting key features under low nitrogen pressure, thus improving the classification performance.

It was clearly evident that the effect of feature selection algorithms gradually improved, with both CARS and Lasso algorithms showing some improvement over the full-band method, but the improvement of single methods was limited. In contrast, the Lasso-CARS algorithm consistently performed the best under both stress conditions, and the degree of improvement increased as the stress level intensified. This highlighted the universality and robustness of the Lasso-CARS algorithm under different stress conditions, significantly enhancing the accuracy and consistency of spectral classification, especially under high-nitrogen stress conditions.

### 2.4. Classification of Nitrogen-Efficient Wheat Varieties at Normal Nitrogen Levels and Selection of Wheat Fertilization Strategies

**Figure 5:**
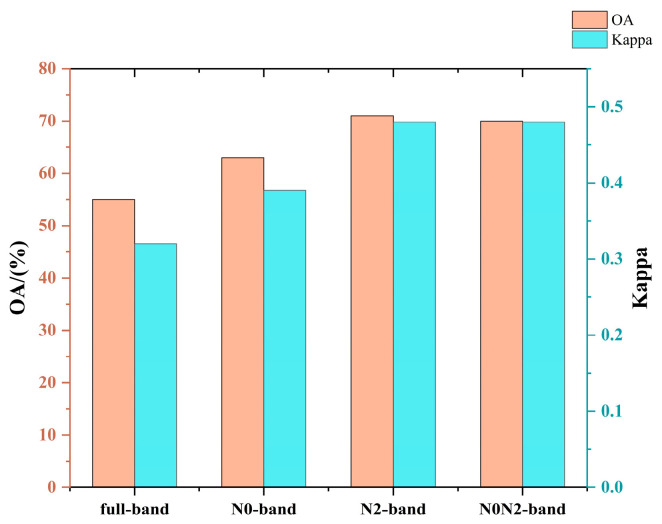
Comparison results of wheat nitrogen-efficient classification models under different feature band combinations as input conditions at normal nitrogen levels. Note: N0 and N2 denote low-nitrogen stress and high-nitrogen stress, respectively. OA denotes the overall accuracy.

According to the classification of nitrogen-efficient wheat varieties under different nitrogen stress conditions, there is no overlap between nitrogen-efficient wheat varieties identified under high-nitrogen and low-nitrogen stress conditions. Therefore, the 12 varieties can be categorized into three groups: low-nitrogen-efficient wheat (including varieties numbered 1, 2, 3, 4, 8, and 12), high-nitrogen-efficient wheat (including varieties numbered 7 and 10), and nitrogen-inefficient wheat (including varieties numbered 5, 6, 9, and 11). Classification models were constructed using the full spectrum, feature bands extracted under low-nitrogen stress, feature bands extracted under high-nitrogen stress, and a combination of both as inputs, with the evaluation results shown in Figure 5.

Among these, the model constructed using the full spectrum performed the worst due to the small differences between nitrogen-inefficient wheat and low-nitrogen-efficient wheat under non-stress conditions, as well as the presence of overlapping bands with those extracted under high-nitrogen stress. Consequently, the classification model constructed using feature bands extracted under low-nitrogen stress alone showed slightly better performance than the full-spectrum approach but was still outperformed by the other two methods. As shown in the figure, feature bands extracted under high-nitrogen stress contributed significantly more to classification accuracy, demonstrating their importance in distinguishing nitrogen-efficient wheat varieties.

Under normal nitrogen levels, there were significant differences in nitrogen absorption and utilization capabilities among different wheat varieties. Based on these differences and their impact on nitrogen fertilizer requirements, appropriate fertilization strategies could be selected for each variety. The classification results of nitrogen-efficient wheat varieties were consistent with the classification results of fertilization strategies. Although nitrogen-inefficient wheat varieties were categorized as “nitrogen-inefficient,” they were more suitable for growth under normal and stable nitrogen fertilizer application conditions compared to the other two nitrogen-efficient types. Maintaining the existing fertilization strategy ensured better nitrogen absorption and utilization efficiency and reduced potential negative impacts on the environment.

Low-nitrogen-efficient wheat varieties exhibited similar nitrogen absorption and utilization efficiency under normal nitrogen application and low-nitrogen stress conditions, and they might perform better when nitrogen supply was limited. However, these varieties were more sensitive to other nutrients, such as phosphorus and potassium. The fertilization strategy could be adjusted by adding small amounts of phosphorus and potassium foliar fertilizers to neutralize excess nitrogen, thereby further optimizing soil nutrient balance and improving yield and quality under normal nitrogen application conditions. Additionally, reducing nitrogen fertilizer application before planting was the most effective fertilization strategy after selecting the wheat variety.

Under high-nitrogen stress conditions, high-nitrogen-efficient wheat varieties demonstrated excellent nitrogen absorption and utilization capabilities, efficiently converting soil nitrogen into biomass and yield. Therefore, increasing nitrogen fertilizer application could further enhance their high-yield potential, especially when nitrogen supply was sufficient. Meanwhile, adopting the nitrogen fertilizer delay technique to postpone nitrogen top dressing until the critical growth stages of wheat (such as the heading stage) could improve nitrogen use efficiency and reduce the risk of nitrogen loss in the early growth stages. This fertilization strategy not only met the high nitrogen requirements of high-nitrogen-efficient varieties but also minimized nitrogen waste through precision fertilization.

## 3. Discussion

### 3.1. Effects of Different Nitrogen Stress Levels on Wheat Phenotype and Agronomic Indices

**Figure 6:**
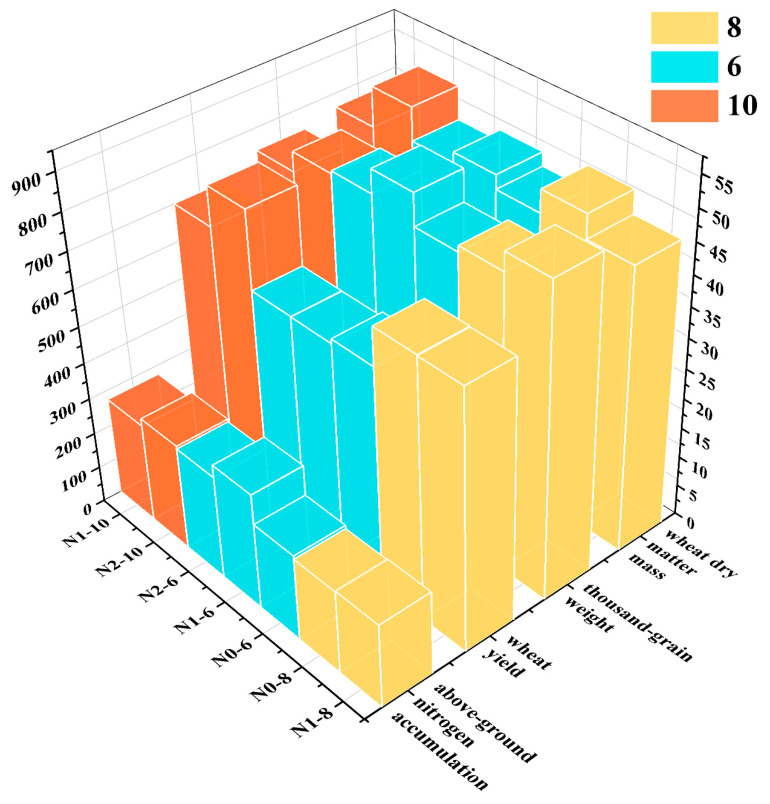
Comparison of phenotypes for different nitrogen-efficient wheat varieties under N0, N1, and N2 nitrogen stress. Note: N0, N1, and N2 denote low-nitrogen stress, normal nitrogen levels, and high-nitrogen stress respectively. Here, 6, 8, and 10 denote a nitrogen-inefficient variety, low-nitrogen-efficient variety and high-nitrogen-efficient variety, respectively.

Wheat growth and development were closely related to nitrogen content in the planting environment. Variations in nitrogen content across planting regions were directly reflected in changes in wheat phenotypes, such as yield, dry matter mass, and nitrogen accumulation. For the same wheat variety, environmental factors could result in distinct phenotypic expressions under different nitrogen conditions. This study focused on key phenotypic traits, including wheat yield, above-ground nitrogen accumulation, thousand-grain weight, and wheat dry matter mass. Using three representative wheat varieties as examples—variety 6 (nitrogen inefficient), variety 8 (low-nitrogen efficient), and variety 10 (high-nitrogen efficient)—the phenotypic variations are illustrated in Figure 6.

For nitrogen-inefficient wheat varieties, both low-nitrogen and high-nitrogen stress significantly and differently affected wheat yield, thousand-grain weight, above-ground nitrogen accumulation, and wheat dry matter mass. As nitrogen is an essential element for synthesizing key biomolecules such as proteins and nucleic acids, its deficiency under low-nitrogen stress conditions inhibited photosynthesis and material accumulation, thereby hindering wheat growth and development [30]. This suppression was particularly evident in the above-ground nitrogen accumulation, thousand-grain weight, and wheat dry matter mass. However, in terms of wheat yield, wheat’s ability to remobilize nitrogen compensated for the deficit. Nitrogen was transferred from older leaves to newer ones, ensuring adequate nitrogen for photosynthesis, which helped maintain physiological processes and growth under low-nitrogen conditions [31]. Conversely, high-nitrogen stress accelerated vegetative growth at the expense of reproductive growth, resulting in reduced wheat yield and thousand-grain weight. Enhanced respiration also consumed more organic matter for maintenance rather than accumulation as dry matter. This nitrogen metabolism imbalance prevented efficient nitrogen absorption, transport, and utilization.

Genetically improved nitrogen-efficient wheat varieties possessed traits that enabled them to maintain high nitrogen absorption and utilization efficiency. These varieties optimized nitrogen uptake and enhanced photosynthetic efficiency to adapt to different nitrogen stress conditions. Under both low- and high-nitrogen stress, nitrogen-efficient wheat showed higher phenotypic values compared to nitrogen-inefficient varieties. In high-nitrogen stress environments, where nitrogen application was increased, nitrogen-efficient wheat exhibited particularly significant improvements in phenotypes such as yield and dry matter mass. In low-nitrogen stress environments, residual nitrogen from previous fertilization regimes may have affected results, leading to modest but noticeable increases in the phenotypes of low-nitrogen-efficient wheat varieties, although the magnitude of improvement was smaller compared to high-nitrogen conditions [32].

### 3.2. Correlation Analysis of Agronomic Indices and Classification of Nitrogen-Efficient Wheat Varieties Under Different Nitrogen Stress Treatments

**Figure 7:**
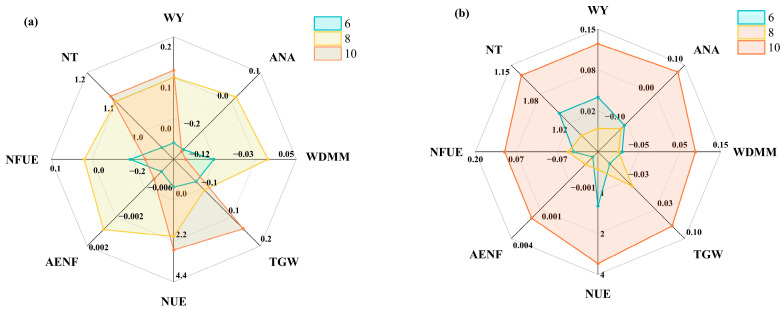
Radar charts of agronomic indices for different nitrogen-efficient wheat varieties. (**a**) Radar chart of agronomic indices under low-nitrogen stress; (**b**) Radar chart of agronomic indices under high-nitrogen stress. Note: WY, ANA, WDMM, and TGW denote the change rates in wheat yield, above-ground nitrogen accumulation, thousand grain weight, and wheat dry matter mass, respectively. UNE, AENF, NFUE, and NT denote wheat nitrogen use efficiency, agronomic efficiency of nitrogen fertilizer, nitrogen fertilizer utilization efficiency, and nitrogen tolerance coefficient, respectively. Here, 6, 8, and 10 denote a nitrogen-inefficient variety, low-nitrogen-efficient variety, and high-nitrogen-efficient variety, respectively. From the data, it was evident that the three wheat varieties—nitrogen-inefficient, low-nitrogen-efficient, and high-nitrogen-efficient varieties—exhibited significant differences across multiple agronomic indices under different nitrogen stress conditions. These differences formed the basis for their classification into distinct groups. Nitrogen-inefficient varieties served as neutral or baseline references, low-nitrogen-efficient varieties optimized nitrogen absorption and utilization in low-nitrogen environments, and high-nitrogen-efficient varieties further enhanced these capabilities under high-nitrogen conditions.

The agronomic indices calculated in this study were influenced by both external factors, such as the planting environment, and internal genetic factors. Different wheat varieties exhibited distinct phenotypes under identical planting conditions due to genotypic differences, leading to variations in agronomic indices. These variations could indirectly reflect nitrogen absorption and utilization efficiency, enabling accurate identification of nitrogen-efficient wheat varieties. Using three representative wheat varieties as examples—variety 6 (nitrogen inefficient), variety 8 (low-nitrogen efficient), and variety 10 (high-nitrogen efficient)—the comparison of agronomic indices for wheat under different nitrogen stress conditions is shown in Figure 7.

On the one hand, nitrogen-efficient wheat varieties exhibited significant yield advantages compared to nitrogen-inefficient varieties. These varieties optimized nitrogen absorption and utilization mechanisms, maintaining high photosynthetic efficiency and material accumulation capacity, thereby ensuring higher yields. TGW was strongly correlated with the classification of nitrogen-efficient wheat, reflecting the distribution of nitrogen to wheat grains [33]. The ANA and WDMM characterized the growth capacity and nutrient production potential of wheat. Nitrogen-efficient wheat varieties enhanced nitrogen absorption efficiency and minimized nitrogen loss, enabling greater nitrogen accumulation. Through rational allocation and utilization, these varieties supported the normal growth and development of above-ground tissues. WY, TGW, ANA, and WDMM directly reflected the trends in reproductive and vegetative growth of wheat under nitrogen stress.

**Figure 8:**
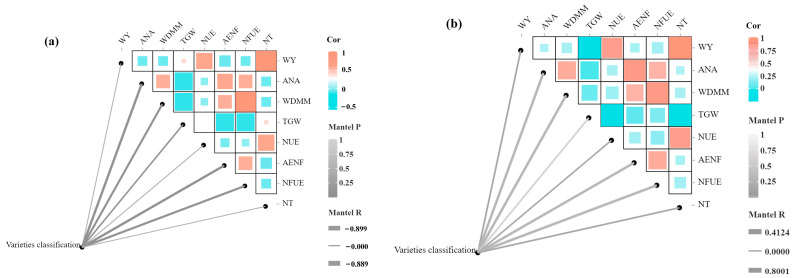
Correlation diagrams between agronomic indices and nitrogen-efficient wheat variety classification (**a**) Correlation diagram under low-nitrogen stress; (**b**) Correlation diagram under high-nitrogen stress. Note: WY, ANA, WDMM, and TGW denote the change rates in wheat yield, above-ground nitrogen accumulation, thousand grain weight, and wheat dry matter mass, respectively. UNE, AENF, NFUE, and NT denote wheat nitrogen use efficiency, agronomic efficiency of nitrogen fertilizer, nitrogen fertilizer utilization efficiency, and nitrogen tolerance coefficient, respectively.

On the other hand, NUE characterized the effect of nitrogen fertilizer on promoting reproductive growth in wheat. Varieties with high nitrogen absorption efficiency responded more significantly to nitrogen fertilizer application, resulting in higher yields [33]. AENF reflected the degree of nitrogen utilization under a given nitrogen application rate. Wheat varieties with high AENF efficiently absorbed nitrogen from the soil, reducing nitrogen fertilizer waste. NFUE measured the proportion of applied nitrogen absorbed and converted into biomass by wheat, indicating the ability to utilize and transform nitrogen fertilizer. NT evaluated the adaptability of wheat to different nitrogen stress conditions based on yield performance. These four agronomic indices collectively reflected the internal processes of nitrogen absorption, utilization, and allocation in wheat. Combining these aspects provided a comprehensive evaluation of nitrogen-efficient wheat varieties. The relationships between agronomic indices and the classification of nitrogen-efficient wheat varieties are illustrated in Figure 8.

The correlations among agronomic indices were generally consistent under different nitrogen stress conditions. However, their relevance to nitrogen-efficient variety classification differed slightly. By comparing the growth and yield performance of different wheat varieties under nitrogen stress and normal conditions, varieties that maintained high economic and biomass yields under nitrogen stress could be identified. These varieties typically exhibited high nitrogen absorption efficiency, achieving greater biomass and grain yields with limited nitrogen resources. Additionally, by measuring NUE, AENF, and NFUE, the responsiveness and utilization efficiency of wheat varieties to nitrogen fertilizer could be further evaluated. This enabled the selection of nitrogen-efficient varieties with high nitrogen fertilizer utilization rates and environmental friendliness. NT was also a crucial indicator for assessing nitrogen efficiency, as it reflected growth performance and yield stability under excessive nitrogen supply.

In conclusion, integrating these indices allowed for the scientific classification and selection of nitrogen-efficient wheat varieties. This provided critical guidance for the rational application of nitrogen fertilizer in agricultural production, ultimately improving wheat yield and quality.

### 3.3. Impact of Different Feature Selection and Classification Methods on Classifying Nitrogen-Efficient Wheat Varieties

In the context of big data, data processing involves a significant amount of irrelevant and redundant information. Extracting extensive data features consumes substantial computational resources, and unsuitable feature subsets could lead to overfitting, low classification accuracy, and other issues. To address the “curse of dimensionality” in big data, it was essential to explore the intrinsic structure and relationships within the data, enhance the distinctiveness and interpretability of variables, and carefully select feature variables [34,35].

This study found that using all hyperspectral bands for classifying nitrogen-efficient wheat varieties yielded suboptimal classification accuracy, required extensive computational resources, and was unsuitable for developing a simple and practical classification model. Thus, constructing the optimal feature set was critical for nitrogen-efficient wheat variety classification. By applying the Lasso-CARS algorithm for hyperspectral feature selection, the results demonstrated improved performance compared to using Lasso or CARS alone, with superior stability. The selected characteristic wavelength ranges were mainly concentrated in the 400–600 nm and 800–1000 nm bands. The 400–600 nm range was primarily associated with pigment absorption, where chlorophyll a/b had significant absorption peaks. Since nitrogen is an essential element for chlorophyll synthesis, the nitrogen content in the canopy leaves of wheat with different nitrogen uptake and utilization efficiencies varied, which in turn affected the content and distribution of chlorophyll. This was reflected in the spectral reflectance [36]. The 800–1000 nm range was associated with carbohydrate absorption. Although it could not directly reflect the nitrogen uptake and utilization efficiency of wheat, this spectral range could reflect information about leaf structure and water content, and it had a significant correlation with the nitrogen content in wheat leaves. Therefore, it could indirectly reflect the nitrogen uptake and utilization efficiency of different wheat varieties [37]. Due to the differences in internal chemical composition among different wheat varieties, these spectral regions contained rich information that could be used to distinguish between different wheat varieties.

Additionally, the analysis revealed certain trends in classification performance. First, classification accuracy under high-nitrogen stress conditions was consistently better than under low-nitrogen stress, regardless of the algorithm used. This indicated that spectral data under high-nitrogen stress have greater discriminatory power and more pronounced feature information. Second, the SVM-XGBoost algorithm, which integrated the strengths of different classification methods, exhibited the best performance under both stress conditions, with particularly notable improvements under high-nitrogen stress. This highlighted its robust feature extraction and classification capabilities. Compared to standalone classification algorithms, SVM-XGBoost significantly improved classification accuracy and consistency by incorporating optimization mechanisms. In contrast, simpler models, such as RF, performed poorly under low-nitrogen stress but improved under high-nitrogen stress, suggesting that the match between spectral features and model complexity was a critical factor influencing classification performance.

Finally, while some algorithms achieved improvements in OA, their Kappa coefficients remained low, indicating insufficient consistency. Therefore, in spectral classification tasks, it was essential to evaluate both OA and Kappa coefficients to comprehensively assess algorithm performance. Additionally, future work will employ ANOVA or paired *t*-tests to compare model performance or classification metrics across models (such as OA and Kappa).

### 3.4. The Advancement of Hyperspectral UAV Remote Sensing Technology in the Classification of Nitrogen-Efficient Wheat Varieties

Traditional methods often employed linear models for classification, which struggled to handle complex multivariate and nonlinear relationships. In contrast, the method proposed in this study comprehensively considered multiple agronomic trait indicators of wheat (wheat yield, above-ground nitrogen accumulation, wheat dry matter mass, thousand-grain weight, agronomic indices) and combined them with hyperspectral feature bands to construct a more comprehensive classification model. Traditional methods for classifying nitrogen-efficient wheat varieties heavily relied on destructive sampling, manual operations, and laboratory analysis, resulting in low classification efficiency. They were primarily suitable for small-scale scientific research trials and variety screening, making it difficult to apply them on a large scale in breeding and production. In contrast, the method proposed in this study achieved rapid, non-destructive data collection using hyperspectral UAV remote sensing technology. Combined with an efficient classification model, it significantly improved classification efficiency and accuracy.

In practical applications of classifying nitrogen-efficient wheat varieties, the proposed method held significant importance for breeders. By regularly inspecting experimental fields with UAVs equipped with hyperspectral cameras during critical growth stages of wheat, breeders could quickly acquire hyperspectral data over large areas and classify varieties. Based on the classification results, breeders could preliminarily screen out nitrogen-efficient varieties, enabling them to focus on the subsequent growth performance of these varieties during the breeding process and significantly accelerate the breeding process. The rapid and large-area coverage capabilities of UAV hyperspectral remote sensing compensated for the temporal and spatial limitations of breeders’ empirical classification methods. For agronomists, the classification results could provide targeted planting advice to growers. They could adjust fertilization strategies according to different wheat varieties, achieving minimal nitrogen fertilizer usage while maintaining high yields, reducing costs, and minimizing environmental impacts.

### 3.5. Future Directions

Hyperspectral UAV remote sensing offers the advantages of high spatial resolution and high flexibility, providing a non-destructive, accurate, and efficient method for field data acquisition and variety classification. However, it also has notable limitations. The acquisition and maintenance costs of hyperspectral sensors and UAVs are relatively high. Hardware equipment such as workstations for processing high-dimensional, large-scale data also needs to be carefully weighed based on actual requirements. The usage fees for data storage and labor costs for processing software also increase with the growing volume of data. These factors, to some extent, limit its application in this field.

Since hyperspectral data can also be obtained from satellites, applying the proposed method for classifying nitrogen-efficient wheat varieties based on satellite remote sensing holds promising prospects [38]. Compared to UAV remote sensing, satellite remote sensing offers broader coverage and does not require frequent flight operations, enabling regular data acquisition over the same area and providing abundant data support [39]. Compared to the large-scale deployment of UAVs, the costs can be amortized over a wider area and a longer period, reducing the cost per unit area [40].

Despite these advantages of applying the method based on satellite remote sensing, it also faces several challenges. The spatial resolution of satellite remote sensing is relatively low, making it unable to capture the subtle features of wheat canopies in individual experimental plots as clearly as UAV remote sensing, which may have a certain impact on the accuracy of the model. Additionally, the acquisition of satellite remote sensing data is constrained by factors such as weather and orbital cycles, resulting in lower data timeliness compared to UAV remote sensing [41]. To address these challenges, data fusion techniques can be employed to organically integrate satellite remote sensing data with UAV remote sensing data [42]. By leveraging the large-scale, long-term sequence information provided by satellite remote sensing data and combining it with the high spatial resolution advantages of UAV remote sensing data, the accuracy and reliability of the classification model can be improved. 

## 4. Materials and Methods

### 4.1. Experimental Design

The wheat experimental field was located at HeFeng Smart Agriculture Base in Zibo City (36°56′33″ N, 118°14′42″ E). This region has a temperate monsoon climate, with an average annual precipitation of 650 mm and an annual average temperature ranging from 12.5 °C to 14.2 °C. The average annual sunshine duration was between 2209.3 h and 2523.0 h. During the 2022–2023 period, 12 nitrogen-efficient wheat varieties bred by Zibo HeFeng Seed Company, as well as the varieties Shannong 28 and Jimai 22, were selected as experimental subjects. Soil nutrient contents in the 0–20 cm soil layer were measured prior to wheat sowing in this experiment, with the following results: organic matter content was 23.6 g/kg, total nitrogen content was 1.420 g/kg, available nitrogen (alkaline hydrolyzable nitrogen) was 113.6 mg/kg, available phosphorus was 21.8 mg/kg, and readily available potassium was 222 mg/kg.

**Figure 9:**
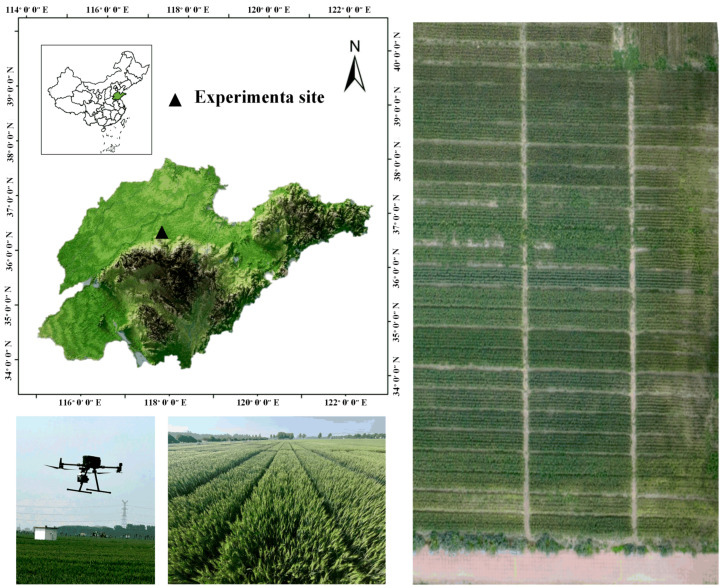
Location of study area and data collection sites. The wheat planting areas subjected to N1, N2, and N0 treatments are shown from left to right.

Based on the local winter wheat planting model (with a standard nitrogen fertilizer application level of 225 kg/ha, where 60% was applied as base fertilizer and 40% was applied as top dressing at the jointing stage, with urea (N ≥ 46%) used as nitrogen fertilizer), three nitrogen fertilizer levels were set: 0 kg/ha (N0), 225 kg/ha (N1), and 450 kg/ha (N2). These levels correspond to low-nitrogen stress, non-nitrogen stress, and high-nitrogen stress, respectively. In this study, ‘high-nitrogen stress’ refers to the physiological effects of excessive nitrogen input, which can hinder wheat growth despite sufficient nutrient availability. Each variety was replicated twice at each nitrogen fertilizer application level. The plots, each with an area of 10 m^2^ (1 m × 10 m), were spaced 20 cm apart, and wheat was planted at a distance of 15 cm. Shannong 28 served as the high-nitrogen stress control group (planted only at N2 and N1 levels), while Jimai 22 served as the low-nitrogen stress control group (planted only at N1 and N0 levels). Fertilization was applied using drip irrigation along with the irrigation water. When fertilizing, it was ensured that all the wheat plants were at the same phenological stage. Phosphorus and potassium fertilizers were applied entirely as base fertilizers in a single application. The experimental field followed uniform planting practices, water and fertilizer management, and pest and disease control. The location and design of the experimental field are shown in Figure 9.

### 4.2. Wheat Phenotype Collection and Agronomic Index Calculation

To ensure the universality and representativeness of the data collected during the wheat phenotyping process, five 0.2 m × 0.2 m areas were designated at the center of each experimental plot and at diagonal positions 4 m away from the center as the sampling ranges for wheat yield and other phenotypic traits. After wheat maturity, one wheat plant was collected from each of the ten areas in total (two experimental plots per variety under the same nitrogen fertilizer application level). Each wheat plant was oven-dried, and its dry matter mass was subsequently measured. The Kjeldahl method was employed to determine the nitrogen accumulation in the wheat ears, stems, and leaves, separately. The total above-ground nitrogen accumulation of the entire wheat plant was calculated by summing these values. The average values of dry matter mass and above-ground nitrogen accumulation were then taken as the corresponding measurements for the variety under the current nitrogen fertilizer application level. Meanwhile, all wheat plants within the ten areas were harvested. After threshing and air-drying, the thousand-grain weight and wheat yield were measured.

The classification of nitrogen-efficient wheat varieties was based on the complex interaction of growth performance, yield traits, and nitrogen use efficiency under different nitrogen levels. Although the indicators used for screening nitrogen-efficient wheat varied due to differences in experimental materials and research priorities, the primary screening metrics remained consistent. Based on comprehensive studies, change rates in wheat yield (WY), above-ground nitrogen accumulation (ANA), thousand-grain weight (TGW), and wheat dry matter mass (WDMM) under nitrogen stress and non-stress conditions were effective indicators of nitrogen absorption and utilization efficiency. The following calculation formula for the change rate was used: (phenotype under nitrogen stress − phenotype under non-nitrogen stress)/phenotype under non-nitrogen stress.

Furthermore, wheat nitrogen use efficiency (NUE), reflecting the ability to absorb soil nitrogen and convert it into wheat yield under a given nitrogen application, was calculated as the ratio of yield differences between nitrogen stress and non-stress conditions to the applied nitrogen. The agronomic efficiency of nitrogen fertilizer (AENF) measured the increase in above-ground nitrogen accumulation per unit of applied nitrogen, while nitrogen fertilizer utilization efficiency (NFUE) represented the growth and development capacity of wheat under varying nitrogen conditions, expressed as the ratio of wheat dry matter mass under nitrogen stress to that under non-stress conditions. The nitrogen tolerance coefficient (NT), expressed as the ratio of wheat yield under nitrogen stress to that under non-stress conditions, indicated wheat’s growth and yield performance under nitrogen-deficient conditions. These agronomic indices were closely related to nitrogen absorption and utilization efficiency and were essential for evaluating wheat nitrogen nutritional status. Therefore, this study classified nitrogen-efficient wheat varieties based on these eight agronomic indicators.

### 4.3. Classification of Nitrogen-Efficient Wheat Varieties

The growth and development of wheat were closely related to the nitrogen content in the planting environment, with phenotypes influenced by both external factors and genetic traits. Wheat of the same variety could exhibit different phenotypes under varying nitrogen levels due to genotypic differences. These phenotypic variations indirectly reflected nitrogen absorption and utilization efficiency, enabling the accurate identification of nitrogen-efficient wheat varieties. Based on the nitrogen content in the environment, planting conditions were categorized into high-nitrogen stress (N2) and low-nitrogen stress (N0) environments.

Under high-nitrogen stress, where fertilizer application was increased, varieties exhibiting significantly higher nitrogen utilization efficiency and phenotypic traits compared to normal nitrogen levels were classified as nitrogen-efficient wheat under high-nitrogen stress. “Shannong 28” was used as the reference variety, and classifications were made into nitrogen-efficient wheat, including “Shannong 28,” and nitrogen-inefficient wheat varieties.

For low-nitrogen stress, reduced nitrogen content led to anticipated lower nitrogen utilization efficiency and phenotypic traits than under normal conditions. Varieties with nitrogen utilization efficiency and phenotypic traits that were equal to or higher than those under normal nitrogen levels were classified as nitrogen-efficient wheat under low-nitrogen stress. Although these varieties may not have shown better performance, they demonstrated superior nitrogen absorption and utilization efficiency compared to others. “Jimai 22” was used as the reference variety, with classifications made into nitrogen-efficient wheat, including “Jimai 22” and nitrogen-inefficient wheat varieties.

After calculating nitrogen efficiency-related agronomic indices and phenotypic traits for different wheat varieties, t-distributed stochastic neighbor embedding (t-SNE) was used for dimensionality reduction. This method reduced the dimensionality of high-dimensional data through probabilistic distribution modeling. First, it computed pairwise similarities between data points in the high-dimensional space based on distances and controlled the scope of local neighborhoods using a perplexity parameter. Next, it initialized random coordinates in the low-dimensional space and recomputed similarities using a t-distribution. Through iterative optimization, it ensured that the low-dimensional embedding preserved both local and global structures of the original high-dimensional data as faithfully as possible. Ultimately, the high-dimensional data were mapped into a two- or three-dimensional space while retaining its local and global structures, thereby providing deeper insights into the underlying data organization. t-SNE excelled particularly in dimensionality reduction for nonlinear data, being capable of better capturing the complex structures within the data. However, its limitations could not be overlooked. First, t-SNE placed excessive emphasis on local structures, which may have led to the loss of global structures, making it difficult to preserve the overall distribution characteristics of the data. Additionally, t-SNE had a high computational complexity and low processing efficiency for large-scale datasets. These limitations may have resulted in deviations in subsequent clustering results. Despite certain limitations of t-SNE, its strengths in capturing local structures, adapting to complex data structures, and providing effective visualization made it more inclined toward classifying wheat varieties.

Hierarchical clustering, known for its flexibility and ability to reveal underlying relationships, was employed due to its suitability for datasets with irregular shapes. The combination of t-SNE and hierarchical clustering was chosen to classify nitrogen-efficient wheat varieties effectively. In dataset preparation, “Shannong 28” and “Jimai 22” were designated as the reference varieties under high- and low-nitrogen stress conditions, respectively, to distinguish classification categories.

### 4.4. Hyperspectral Data Collection

To obtain stable spectral reflectance data of the winter wheat canopy, a UAV flight experiment was conducted during the wheat heading stage (27 April) under clear and cloudless weather conditions between 11:00 and 13:00. The experiment utilized an M300 RTK UAV (Da-Jiang innovations science and technology Co., Ltd., Shenzhen, China) equipped with a GaiaSky-mini3 hyperspectral imaging system (Jiangsu Dualix Spectral Imaging Technology Co., Ltd, Wuxi, China) developed by Dualix, with a spectral range of 400–1000 nm, a spectral resolution of 5.5 nm, and an image resolution of 1024 × 1000 pixels.

Before the UAV took off, the exposure time was automatically set, and the calibration plate and blackboard data were collected for reflectance correction and dark current correction. The drone flew at a height of 50 m and a speed of 5 m per second and hovered at a preset waypoint to collect hyperspectral data of the wheat canopy. At the same time, the calibration plate data at the same height were collected during each data acquisition for later atmospheric data correction. The acquired hyperspectral data underwent lens correction, reflectance calibration, atmospheric correction, and radiometric calibration to convert the original digital number values into reflectance. The corrected images were mosaicked and stored in TIFF format. Preprocessed remote sensing images were then clipped using ArcGIS 10.8 software to obtain the spectral images of each planting plot. Based on the location and shape characteristics of the planting plots in the experimental wheat field, the center of each plot was determined. A 20 × 20-pixel region was selected at the center and at one-fifth of the distance from each vertex to the center. Spectral reflectance values corresponding to each plot were extracted from these regions.

### 4.5. Development of Classification Model for Nitrogen-Efficient Wheat Varieties

#### 4.5.1. Selection of Hyperspectral Characteristic Bands

The collected canopy hyperspectral data of wheat involved a large volume of information, often influenced by noise and high homogeneity within similar wavelength ranges. Before constructing a model, it was essential to filter the hyperspectral data to remove redundant and highly correlated bands while retaining significant characteristic bands. This approach enhanced model accuracy and reduced computation time. To address these issues, this study proposed a feature selection strategy combining least absolute shrinkage and selection operator (Lasso) and competitive adaptive reweighted sampling (CARS).

Lasso, a sparse constraint-based linear regression method, offered good interpretability and effectively handled multicollinearity in high-dimensional data by introducing an L1 regularization constraint. This approach eliminated coefficients of irrelevant or highly collinear bands, thereby isolating essential characteristic bands relevant to the target variable. Consequently, Lasso served as the primary method for spectral band selection.

CARS further optimized the bands identified using Lasso by simulating variable importance analysis in partial least squares regression. Through random sampling and adaptive reweighting, CARS progressively eliminated redundant and noisy bands, refining the feature set to contain more informative key bands. Lasso’s robustness enhanced feature selection accuracy and stability, providing high-quality candidate sets for subsequent optimization. Meanwhile, CARS’s mechanism focused on identifying the most significant bands, thereby improving accuracy and robustness in feature selection.

By combining Lasso and CARS, the method addressed the high dimensionality, redundancy, and strong correlation in hyperspectral data. It preserved the integrity of critical band information, improving the generalization performance and accuracy of the classification model. Additionally, this approach minimized interference from noise and redundant variables, simplified model complexity, and reduced data dimensionality. Compared to single-feature selection methods, the Lasso-CARS strategy excelled in computational efficiency and robustness, making it an efficient and reliable solution, especially in complex applications such as hyperspectral remote sensing.

#### 4.5.2. Development of the Classification Model for Nitrogen-Efficient Wheat Varieties

Support vector machine (SVM) was a widely used algorithm for classification tasks, particularly suited for high-dimensional and small-sample feature data. The core idea of SVM was to map input features into a higher-dimensional space using kernel functions, thereby identifying an optimal decision boundary that maximized the margin between different classes while minimizing classification errors. SVM focused on “support vectors”—critical sample points located near the decision boundary—to define the hyperplane, ensuring the model’s robustness against outliers and noise. For nonlinear problems, SVM implicitly computed dot products in the high-dimensional space, avoiding explicit dimensional transformations and thus enabling efficient classification. In the classification of nitrogen-efficient wheat varieties, feature data often included multidimensional physiological, environmental, and genetic factors with highly nonlinear and complex relationships. SVM effectively addressed these complexities. Its built-in regularization mechanism also prevented overfitting, enabling SVM to extract distinguishing features from complex feature spaces and build reliable classification boundaries.

However, while SVM performed well in many classification tasks, it may have faced challenges when dealing with highly non-linear data, especially in high-dimensional datasets with complex interactions and noise. In such scenarios, the ensemble learning method based on gradient boosting decision trees, such as gradient boosting decision tree (GBDT) and extreme gradient boosting (XGBoost), exhibited better performance. It trained multiple decision trees iteratively. Each new tree focused on learning the prediction error of the previous tree, and the overall performance was improved by weighting and combining the output results of all trees. This approach gradually optimized the model’s ability to fit data and then modeled complex nonlinear relationships. It could automatically capture the nonlinear relationships and interactive effects between features and exhibited robustness to noise. This made it particularly effective in dealing with the subtle and complex patterns present in wheat canopy spectral data, thereby complementing the advantages of SVM.

**Figure 10:**
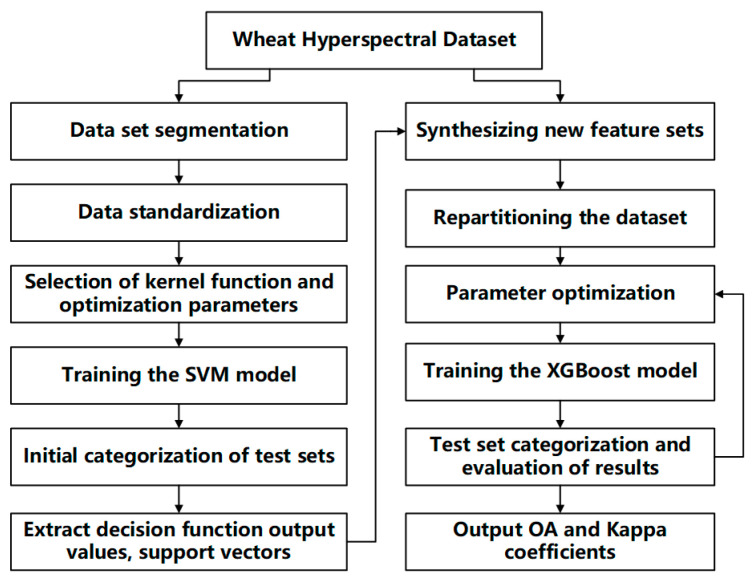
Flowchart of the SVM-XGBoost approach.

To accurately identify nitrogen-efficient wheat varieties, this study proposed a serial hybrid classification method integrating SVM and XGBoost. The method combined SVM’s strong boundary construction capability with XGBoost’s superior nonlinear modeling performance. The workflow is illustrated in Figure 10.

First, the SVM model processed complex decision boundaries with its adaptability to high-dimensional features, particularly in scenarios involving nonlinear relationships between features and categories. During training, SVM constructed a decision hyperplane to maximize inter-class margins, efficiently identifying key features distinguishing nitrogen-efficient and inefficient wheat varieties. This process was initiated with dataset segmentation and data standardization, followed by the selection of kernel function and optimization parameters, and culminating in the training of the SVM model. As depicted in the flowchart, the initial categorization of test sets was then performed, and decision function output values along with support vectors were extracted.

Next, outputs from the SVM model, including decision function values and support vector information, were used as additional features for input into the XGBoost model. Leveraging XGBoost’s powerful ensemble learning capabilities and adaptability to nonlinear relationships, the model employed gradient boosting trees to refine the classification model further and improve its performance. This part of the process involved synthesizing new feature sets, repartitioning the dataset, and optimizing parameters before training the XGBoost model. The test set categorization and evaluation of results were then conducted to assess the model’s accuracy.

By complementing the strengths of both SVM and XGBoost, the serial fusion model achieved higher accuracy and stronger generalization capabilities in the classification of nitrogen-efficient wheat varieties. Compared to single models, the serial fusion of SVM and XGBoost not only overcame issues such as overfitting or underfitting that may have occurred with individual models, but also fully leveraged the strengths of each model. Specifically, SVM excelled in handling high-dimensional features and small sample sizes, while XGBoost efficiently addressed nonlinear relationships in the data. Given that the classification of nitrogen-efficient wheat varieties involved complex multi-dimensional physiological traits and environmental factors, this fusion method was better able to capture these intricate relationships, thus improving classification accuracy and making it suitable for practical field breeding trials in wheat nitrogen efficiency classification.

#### 4.5.3. Accuracy Evaluation

Ten-fold cross-validation served as the method for dataset partitioning. Ten-fold cross-validation is a commonly used model validation technique aimed at evaluating the generalization performance of the model. Ten-fold cross-validation was primarily applied to small sample datasets, where the average value was ultimately selected as the final performance metric of the model. The overall accuracy (OA) and Kappa coefficient were employed to assess the accuracy of the model (As shown in Equations (1) and (2)). OA referred to the ratio of the number of correctly classified samples to the total number of samples, measuring the overall classification accuracy of the model across the entire dataset. A higher value indicated better classification performance of the model on the dataset, with a higher proportion of correct classifications. The Kappa coefficient was an index used to measure the consistency of classification model performance. It considered the degree of agreement between the model’s predictions and the actual classification results. The Kappa coefficient provided a more comprehensive reflection of the model’s classification performance, especially when dealing with imbalanced datasets. It offered a more objective assessment of the model’s performance across various categories. A higher Kappa coefficient signified better agreement between the model’s predictions and the actual classification results, indicating more reliable classification performance of the model. The cross-validation was stratified by nitrogen level and nested within each treatment group.

(1) $$OA=\frac{\sum_{i=1}^{n}x_{ii}}{N}\times 100\%$$

(2) $$Kappa=\frac{N\sum_{i=1}^{n}x_{ii}-\sum_{i=1}^{n}x_{i+}x_{+i}}{N^{2}-\sum_{i=1}^{n}x_{i+}x_{+i}}$$

## 5. Conclusions

This study validated the effectiveness of drone remote sensing hyperspectral technology in classifying nitrogen-efficient wheat varieties. Based on the clustering results of agronomic indices and canopy hyperspectral data, the Lasso-CARS method was employed to extract characteristic band combinations relevant to the classification of nitrogen-efficient wheat. A classification model for nitrogen-efficient wheat varieties was then constructed using the proposed SVM-XGBoost algorithm. The model built on the characteristic band combinations achieved an average OA of 76% and an average Kappa coefficient of 0.65, enabling efficient and accurate classification of nitrogen-efficient wheat varieties. This approach addresses the issues of high labor intensity, long time requirements, and low efficiency associated with traditional classification methods for nitrogen-efficient wheat varieties.

The proposed method has demonstrated its practicality in field breeding environments, providing valuable technical support for streamlining the wheat breeding process. It represents an efficient and accurate method for classifying nitrogen-efficient wheat varieties. Furthermore, to ensure the widespread applicability and stability of the proposed method, we plan to expand its application scope to include datasets covering a broader range of wheat varieties. This will enhance the generalization ability of the method and further ensure its reliable performance in the breeding process.

## Figures and Tables

**Table 1:** Statistics of agronomic indices under low-nitrogen stress.

	Agronomic Indices	WY	ANA	WDMM	TGW	UNE	AENF	NFUE	NT
Item									
Max		10%	−1%	11%	17%	2.68	−0.0002	0.098	1.095
Min		−8%	−30%	−25%	−18%	−2.597	−0.009	−0.349	0.918
SD		0.0515	0.105	0.096	0.0978	1.445	0.003	0.118	0.051
Mean		−0.004	−0.173	−0.115	−0.008	−0.149	−0.005	−0.145	0.996
CV		13.37%	0.60%	0.83%	11.94%	9.72%	0.66%	0.81%	0.05%

Note: max, min, SD, mean, and CV denote maximum value, minimum value, standard deviation and coefficient of variation, respectively. WY, ANA, WDMM, and TGW denote the change rates in wheat yield, above-ground nitrogen accumulation, wheat dry matter mass, and thousand grain weight, respectively. UNE, AENF, NFUE, and NT denote wheat nitrogen use efficiency, agronomic efficiency of nitrogen fertilizer, nitrogen fertilizer utilization efficiency, and the nitrogen tolerance coefficient, respectively.

**Table 2:** Statistics of agronomic indices under high-nitrogen stress.

	Agronomic Indices	WY	ANA	WDMM	TGW	UNE	AENF	NFUE	NT
Item									
Max		13%	23%	24%	14%	3.557	0.005	0.213	1.12
Min		−10%	−12%	−10%	−12%	−3.133	−0.004	−0.121	0.900
SD		0.059	0.109	0.109	0.083	1.761	0.003	0.111	0.059
Mean		−0.006	0.006	0.019	0.004	−0.243	−0.00013	0.011	0.994
CV		10.43%	17.08%	5.56%	19.29%	7.25%	20.00%	10.21%	0.06%

Note: max, min, SD, mean, and CV denote maximum value, minimum value, standard deviation and coefficient of variation, respectively. WY, ANA, WDMM, and TGW denote the change rates in wheat yield, above-ground nitrogen accumulation, wheat dry matter mass, and thousand grain weight, respectively. UNE, AENF, NFUE, and NT denote wheat nitrogen use efficiency, agronomic efficiency of nitrogen fertilizer, nitrogen fertilizer utilization efficiency, and the nitrogen tolerance coefficient, respectively.

**Table 3:** Comparative experiment results of different models.

	Models	SVM-XGBoost		SVM		RF		XGBoost		Adaboost	
Nitrogen Situation		OA (%)	Kappa	OA (%)	Kappa	OA (%)	Kappa	OA (%)	Kappa	OA (%)	Kappa
Low-nitrogen stress		74	0.67	55	0.55	65	0.41	69	0.38	65	0.40
High-nitrogen stress		83	0.80	79	0.79	76	0.68	76	0.74	79	0.72
Mean		78.5	0.74	67	0.67	70.5	0.55	72.5	0.56	72	0.56

Note: SVM, XGBoost, RF, and Adaboost denote support vector machine, extreme gradient boosting, random forest, and adaptive boosting, respectively. OA denotes the overall accuracy.

**Table 4:** Comparative experiment results of different subsets feature bands inputs.

	Bands	Full Band		CARS		Lasso		Lasso-CARS	
Nitrogen Situation		OA(%)	Kappa	OA(%)	Kappa	OA(%)	Kappa	OA(%)	Kappa
Low-nitrogen stress		65	0.35	69	0.52	65	0.51	74	0.67
High-nitrogen stress		73	0.67	80	0.74	79	0.73	83	0.80
Mean		69	0.51	74.5	0.63	72	0.62	78.5	0.74

Note: CARS and Lasso denote competitive adaptive reweighted sampling and least absolute shrinkage and selection operator, respectively. OA denotes the overall accuracy.

## Data Availability

Data will be made available on request.
